# Effect of valerian on cognitive disorders and electroencephalography in hemodialysis patients: a randomized, cross over, double-blind clinical trial

**DOI:** 10.1186/s12882-018-1134-8

**Published:** 2018-12-27

**Authors:** Afshin Samaei, Monir Nobahar, Zaynab Hydarinia-Naieni, Abbas Ali Ebrahimian, Mohammad Reza Tammadon, Raheb Ghorbani, Abbas Ali Vafaei

**Affiliations:** 1 Research Center and Department of Physiology, Faculty of Medicine, Semnan University of Medical Sciences, Semnan, Iran; 2 Nursing Care Research Center and Social Determinants of Health Research Center, Faculty of Nursing and Midwifery, Semnan University of Medical Sciences, Semnan, 3513138111 Iran; 3 Department of Nursing, Faculty of Nursing and Midwifery, Semnan University of Medical Sciences, Semnan, Iran; 4 Nursing Care Research Center, Semnan University of Medical Sciences, Semnan, Iran; 5 Kowsar Hospital, Semnan University of Medical Sciences, Semnan, Iran; 6 Social Determinants of Health Research Center, Department of Epidemiology and Biostatistics, Faculty of Medicine, Semnan University of Medical Sciences, Semnan, Iran; 7 Rehabilitation Research Center, Neurology Department, Kowsar Hospital, Faculty of Medicine, Semnan University of Medical Sciences, Semnan, Iran

**Keywords:** Cognitive Disorders & Electroencephalography, Hemodialysis, Valerian

## Abstract

**Background:**

The prevalence of cognitive disorders in hemodialysis patients is twice as high as the general population, while these disorders often are undiagnosed. Timely prevention and treatment can improve their personal and social functions. Aim of study was determined the effect of Valerian on cognitive disorders and electroencephalography (EGG) in hemodialysis patients.

**Methods:**

This crossover, double-blind clinical trial was conducted on 39 hemodialysis patients. The patients were randomly divided into two groups. Group A (*n* = 19) took Valerian capsules and Group B (*n* = 20) received placebo capsules 60 min before bedtime for one month. The type of treatment was replaced between the two groups after a one-month wash-out. The Mini Mental State Examination (MMSE) questionnaire was completed and EGG was performed before and after intervention in both periods.

**Results:**

The cognitive scores of the Group valerian were increased significantly in the first (*p* = 0.003) and the second (*p* = 0.005) periods. In addition, the mean increase in the cognitive scores in the Group valerian was significant in the first (*p* = 0.028) and the second periods (*p* = 0.030). However, the changes in EGG showed no significant difference before and after intervention in two groups.

**Conclusion:**

The findings of this study indicated that valerian could be effective and significantly improve patients’ cognitive status; however, no significant changes were observed in the electroencephalography of the hemodialysis patients.

**Trial registration:**

IRCT201606076318N7–2016-06-17.

## Background

The prevalence of cognitive disorders in the hemodialysis patients is twice as high as in the general population [1], The prevalence of cognitive disorders in the Dialysis Population from 6.6 to 51% [2]. which occur frequently in the hemodialysis patients due to several factors, including serum level of lipids, low level of education, race [3] and aging [4, 5]. The risk of cognitive disorders can be increased because of vascular diseases and the high prevalence of chronic diseases such as diabetes, hypertension, anemia [6], abnormal sleep [7] and acute vascular events like strokes [8]. Following the stroke, the risk of dementia and cognitive disorders are up to 9 times more common [8]. Type II diabetes is associated with a decrease in cognitive function, especially in verbal memory, information processing speed and executive functioning [9]. However, the hemodialysis, in turn, has no significant effect on the overall brain function [10].

Since, no specific screening tool can cover all the requisite cognitive domains nor have any instrument been specifically validated for hemodialysis patients against a clinical diagnosis of cognitive disorders [11], Mini-Mental State Examination (MMSE) as the most commonly used screening tool for cognitive disorders has been of use in numerous studies [11–15] and it has been also well-known to clinicians [11]. The use of MMSE is one of the common methods for detecting cognitive disorders, but this questionnaire alone is not enough to examine cognitive status [16]. In other words, the normal function and score in this questionnaire do not rule out cognitive impairment, because individuals with MMSE≥24 also have a high frequency of poor cognitive functioning [16].

The electroencephalography (EEG) is an extremely beneficial, non-invasive and relatively inexpensive method to investigate the damage to the level of consciousness, the confusion scenarios, acute and sub-acute cognitive problems, which is considered as an irreplaceable procedure in the diagnosis and management of cerebral cortex disorders [17]. The methods of EEG signal coupling and synchronization can also play a key role in evaluating and diagnosing patients with mild cognitive impairment (MCI) [18]. Besides, many studies have demonstrated the initial value of the coupling and synchronization analysis of EEG signals with applications for evaluating MCI [19–22]. Studies have also shed light on the use of synchronization likelihood for analyzing EEG signals in MCI patients [23]. The EEG changes are detected in patients compared to normal and are considered as a control method for treatment interventions [24]. In fact, the hemodialysis patients experience cognitive impairment and subsequently EEG changes, emphasizing the reversibility of memory changes in these patients, so that these disorders can be quickly resolved with early diagnosis [25], while these abnormalities often are undiagnosed or overlooked. Their timely prevention and treatment will result in improved personal and social functioning. [15, 26, 27]. Kallenberg et al. (2016) reported that understanding these associations could ultimately lead to prediction models to guide tailored treatment decisions or preventive interventions [26]. In this regard, the results of the study by Bossola et al. (2011) suggested the importance of strict monitoring of cognitive functions in end-stage renal disease patients receiving chronic hemodialysis and provided evidence that the development of adequate strategies for the prevention and treatment of cognitive impairment was of priority [28]. Despite the absence of evidence-based cost-effective therapies for cognitive disorders, detecting of this treatment in this population was supposed to create an opportunity to proactively personalize care through education, support decision-making, and also adopt targeted communication strategies in order to cover specific areas of deficits and consequently help in defining care-related goals [11].

Complementary medicine has attracted further attentions among the various approaches to heal cognitive disorders. The name valerian, *Valeriana officinalis*, comes from the Latin word valere, meaning to be strong or healthy [29]. Valerian contains 150 to 200 different substances, including volatile oils, ketones, and phenols, iridoid esters such as valreotriate, valric acid, alkaloids, and amino acids like aminobutric acid, tyrosine, arginine, glutamine and noncyclic, monocyclic and bicyclic hydrocarbons [30]. This herbaceous perennial plant with short rhizomes creates underground creeping stem and is widely found in temperate regions of Asia, Europe and North America, and has beneficial properties for the heart, brain and stomach [31].

Valerian extract as an agonist of adenosine A1 receptors inhibits cholinergic transmission, increases the frequency strength of delta, theta and alpha waves in the frontal cortex and has sedative-like effects [32]. Valerian has been considered as a sedative in Europe and then the United States since the 16th and 17th centuries [33] and has been a part of the pharmacopoeia in Europe and America [34]. The American Herbal Products Association (AHPA) categorized Valerian as Class I in terms of health and safety, and the US Food and Drug Administration has allowed its entry in food [35]. Valerenic acid, as a sesquiterpenoid, is considered among the major secondary bioactive metabolites of *Valeriana officinalis* L. Until now, the number of in vivo studies on the absorption, bioavailability, disposition, and metabolism of Valerenic acid has been limited. Pharmacokinetics of Valerenic acid in rats after oral treatment has been also described by a two-compartment model with a clearance (CL/F) of 2–5 L/h/kg and a volume of distribution of 17–20 L/kg. The extent of the absorption after oral administration has been similarly estimated to be 33.70% with a half-life of 2.7–5 h [36]. According to a study on valerian, different doses of this medication could cause no increase in blood urine nitrogen and creatinine (compared to sham group). Therefore, the extract of this herb was not likely to have toxic effects on rat kidneys [37].

Scientific studies on valerian have begun on humans since 1970 [33]. Vonderheid-Guth et al. (2000) argue that the use of valerian-hops mix has pharmacodynamic responses in the brain [38]. Schulz et al. (1998) in two multiple crossover studies, each involving 12 adult female subjects, screened for acute sedative effects of eight different plant extracts. Valerian extract, which was administered in both studies, displayed an increase of power in the delta and theta bands and a decrease in the beta band. The results correspondingly showed that sedating effects of plant extracts could be identified by quantitative EEG analysis as well as self-assessment instruments [39]. In addition, Hasani et al. (2013) has suggested valerian as a prophylactic strategy for the prevention of cognitive disorders after heart surgery [40].

Since cognitive disorders are common in hemodialysis patient, the severity and characteristics of them are not well defined, which are associated with prolonged hospitalization, poor quality of life, mortality and morbidity among the hemodialysis patients [4, 41]. On the other hand, cognitive disorders have more serious consequences; for example, lowering life expectancy that might prevent the hemodialysis patients participating in the hemodialysis programs, taking medications and adhering to dietary restrictions [42]. Therefore, it is essential to investigate the cognitive disorders and control the related complications in order to achieve better therapeutic outcomes in these patients. Considering the effects of valerian extract on brain wave in EEG and its possible role in improving cognitive disorders, we decided to evaluate the effect of valerian on cognitive disorders and EEG in the hemodialysis patients.

## Methods

This crossover clinical trial was a double-blind study conducted on 39 hemodialysis patients in Semnan and Mahdishahr hospitals in 2016, who met the inclusion criteria. In a preliminary study, the mean scores ± standard deviation of the cognitive changes were calculated for two groups of 10 patients, before and after the intervention in group A was 1.27 ± 1.21 and in the group B was 0.29 ± 0.92. Then, the following equation was used to calculate the sample size. Considering 95% confidence and 80% power, the maximum sample size was obtained as 19 patients per group.

$$ n={\frac{\left({S}_1^2+{S}_2^2\right)\times \left({Z}_{1-\frac{\alpha }{2}}+{Z}_{1-\beta}\right)}{{\left({\overline{X}}_1-{\overline{X}}_2\right)}^2}}^2 $$

The inclusion criteria were aged over 18 years, hemodialysis treatment three times a week for four hours, a history of hemodialysis for at least three months. The exclusion criteria involved a physical disability or a certain mental disorder, drug addiction, cancer, hearing or visual impairments preventing proper communication, experience the latest stressful event, such as pregnancy, kidney transplantation during the study, acute illness or acute renal failure, body mass index over thirty (BMI > 30), history of liver disease, hepatitis, cirrhosis, travel or death.

The data collection tools were demographic information, MMSE questionnaires and EEG. The demographic information of patients was asked after obtaining consent. Folstein and McHugh (1975) introduced the MMSE that is the most common instrument for cognitive screening suitable for global use. The MMSE consists of 11 questions with score of 30 points, including 16 points for memory and orientation, 5 points for attention and concentration, 8 points for assessing the language and understanding abilities, and 1 point for visual-spatial abilities; the total scores of 25–30 for health, 21–24 for mild, 10–20 for moderate and less than 9 for severe cognitive disorders [43].

The EEG was taken with the NeoFax device (Nihonkohden Co., Japan), a bipolar 10/20 equipment with 23 electrodes [17]. The same neurologist interpreted the EEG, according to the types and frequencies of different brain waves. The waves in almost normal range were usually alpha (8–13 Hz per second) and beta (over 13 Hz per second), the waves with frequencies less than 8 (delta: 1 to 3 and theta: 4 to 7 Hz per second) were abnormal.

**Fig 1.**
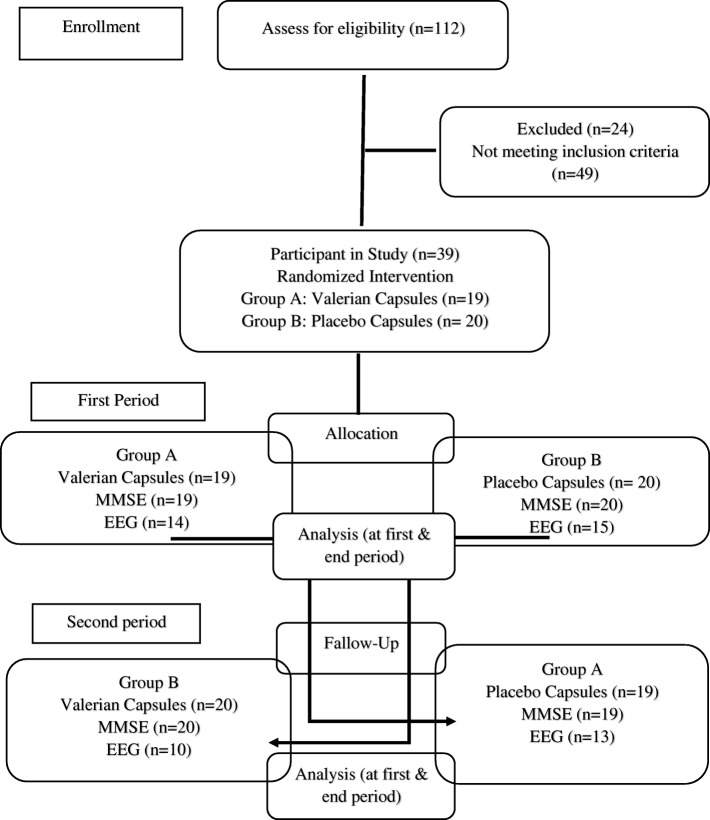
Flow chart of the study design, enrollment, randomization, follow-up and analysis of study participants

**Fig 2:**
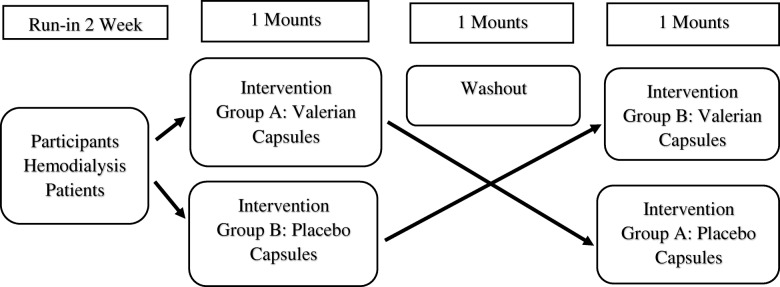
Study Schema

The patients were randomly divided into two groups using a coin toss. The first patient entered group A if the coin was seen and the patient was placed in group B if the tails was shown. The next patient, who was similar in terms of gender and age difference of ±5 years, was also assigned to the opposite group, this process continued until to reach the sample size. The crossover methods could cause both groups A and B receive valerian and placebo capsules. Before the intervention, the MMSE of the patient was completed and the EEG was performed. Given that the patients were undergoing hemodialysis in different shifts (morning, evening, and night), the MMSE was completed in all three shifts at the beginning of the hemodialysis procedure; duo to limit participant fatigue, testing was completed the first hour of hemodialysis and then the EEG was done in the morning shift on the day after hemodialysis between 8 AM until 12 MD (Fig. 1). In this study, the use of valerian and placebo capsules was supervised by a nephrologist as the only person aware of the type of intervention. The study was double-blind, participants and investigator as well as statistician were blind to the study groups until the analysis was completed. Then, the Group A received Valerian capsules (Sedamin 530 mg, Goldaru Co.) and Group B took placebo capsules (Starch 50 mg, Goldaru Co.) 60 min before bedtime for one month. After a one-month wash-out, the drug regimen was replaced between the two groups, as placebo capsules for Group A and valerian capsules for Group B (Fig. 2). The valerian and placebo capsules were coated with the same color. The EEG was taken at baseline and end of both phases of intervention, and MMSE was evaluated. The patients were requested to report any problem with the drug to the researcher, and were ensured that they can leave taking medication whenever they want. Regular use of capsules and possible side effects weekly was followed up by telephone and on-line visits to hemodialysis centers.

The data were analyzed in SPSS 18 software using Kolmogorov-Smirnov, Chi-square, McNemar’s, T-test, Mann-Whitney, Paired t-test or Wilcoxon tests at a significance level of 0.05 (Fig. 2).

## Result

The mean ± SD of patients age was 66.4 ± 14 years in group A and 65.6 ± 12.4 years in group B; the difference was not significant (*P* = 0.857). Minimum and maximum ages were respectively 35 and 88 years in group A and 41 and 86 years in group B. Females consisted of 52.6% of patients in group A and 45.0% of patients in group B; the difference was not significant (*P* = 0.634). The mean BMI was 23.6 ± 3.3 kg/m^2^ in group A and 23 ± 3.1 kg/m^2^ in group B; the difference was not significant (*P* = 0.549). None of the patients in both groups was obese (BMI ≥ 30). All patients in both groups were married, 31.6% in group A and 35% of patients in group B were illiterate. Distribution of patients’ literacy level was not significantly different between the two groups (*P* = 0.588). The level of income was low in 21.1% of patients in group A and 30.0% of patients in group B. Distribution of income between the two groups were not significantly different (*P* = 0.513). None of the patients in group A was smoker and only 1 patient (5%) in group B was smoking; the difference was not significant (*P* = 1.00). Diabetes mellitus was the most common cause of dialysis in both groups; the difference was not significant (*P* = 0.618). In this respect, 26.3% of the patients in group A and 25% of them in group B had heart disease and the difference was not significant (*p* = 0.925). Moreover, 68.4% of the individuals in group A and 45.0% of them in group B suffered from restless leg syndrome (RLS) although the difference was not significant (*p* = 0.140). In terms of taking tea, distribution of cups consumed was not significantly different between the two groups (*P* = 0.857) (Table 1). As well, 68.4% of the patients in group A and 75% of them in group B had a history of hemodialysis of less than 5 years. The mean ± SD of the duration of dialysis in group A patients was also equal to 3.42 ± 2.75 years and they were 3.55 ± 2.96 years in group B patients. The duration of hemodialysis in both groups was not significant (*p* = 0.945). Duration of hemodialysis in each dialysis session was 4 h in all patients in both groups. In this regard, 26.3% of the patients in group A and 30.0% of them in group B had vascular fistula although the difference was not significant (*p* = 0.998). Other patients in both groups were also undergoing hemodialysis with long-term hemodialysis catheter (permcath). None of the patients in group A had a history of lung disease and 10% (*n* = 2) of patients in group B had a history of lung disease; the difference was not significant (*P* = 0.487). None of the patients had a history of gastrointestinal disease groups. Moreover, 52.6% (*n* = 10) of patients in group A and 35% (*n* = 7) of patients in group B were taking hypnotic drugs; the difference was not significant (*P* = 0.267). Anti-anxiety and anti-depression drugs were not reported in any of the patients in both groups.

**Table 1:** Participant’s characteristics in the groups under study

Index		Group			
		A^a^		B	
		Number	Percent	Number	Percent
Sex	Female	10	52.6	9	45
	Mal	9	47.4	11	55
Body Mass Index	> 18.5	1	5.3	3	15
	18.5–24.9	12	63.2	12	60
	25–29.9	6	31.6	5	25
Education level	illiterate	6	31.6	7	35
	Elementary	9	47.4	6	30
	Diploma or higher	4	21.1	7	35
Income	Low	4	21.1	6	30
	Average	14	73.7	14	70
	Good	1	5.2	–	–
Dialysis causes	DM	7	36.8	5	25
	HTN	4	21.1	4	20
	DM,HTN	4	21.1	8	40
	Other	4	21.1	3	15
HD	+	5	26.3	5	25.0
	–	14	73.7	15	75.0
RLL	+	13	68.4	9	45/0
	–	6	31.6	11	55.0
Number of cups of tea	0	–	–	2	10
	1	5	26.3	5	25
	2	13	68.4	10	50
	≥3	1	5.3	3	15

^a^The group A took valerian capsules in the first therapeutic period of one month and placebo in the second therapeutic period of one month, and vice versa in group B

The total score of MMSE in the Group valerian was increased significantly in the first month (*P* = 0.003) and the second month (*P* = 0.005) of treatment. The scores of concentration and calculation subscales in the Group valerian were increased significantly in both periods (*P* = 0.014). Such an increase was also observed in the memory subscale in the first one-month (*P* = 0.014) and the second one-month (*P* = 0.46) periods. In other subscales, no significant increase was seen in any of the groups (Table 2).

**Table 2:** Mean and SD of cognitive scores groups A and B in first and second periods

Index	group	First Period						Second Period							
		Before intervention			After intervention				Before intervention			After intervention			
		N	Mean	SD	N	Mean	SD	P-value	N	Mean	SD	N	Mean	SD	*P*-value
Orientation	A^a^	19	9.68	0.58	19	9.95	0.23	0.059	17	9.94	0.24	17	9.94	0.24	1.00
	B	20	9.50	1.10	20	9.60	1.00	0.157	20	9.60	1.05	20	9.85	0.27	0.101
	P-value	–	0.923		–	0.428			–	0.619		–	0.641		
Immediate memory	A^a^	19	2.95	0.23	19	3.00	0.00	0.317	17	3.00	–	17	3.00	0.00	1.00
	B	20	2.75	0.44	20	2.85	0.37	0.157	20	2.75	0.64	20	2.80	0.62	0.317
	P-value	–	0.296		–	0.428		–	–	0.442		–	0.619		
Concentration and calculation	A^a^	19	3.58	1.22	19	4.05	1.08	0.014	17	3.71	1.10	17	3.71	1.26	1.00
	B	20	3.75	1.25	20	3.75	1.19	1.00	20	3.75	1.37	20	4.05	1.19	0.014
	P-value	–	0.708		–	0.531		–	–	0.940		–	0.424		–
Remembrance	A^a^	19	2.53	0.61	19	2.84	0.37	0.014	17	2.59	0.51	17	2.65	0.49	0.317
	B	20	2.40	0.68	20	2.40	0.75	1.00	20	2.35	0.74	20	2.55	0.69	0.046
	P-value	–	0.627		–	0.095		–	–	0.442		–	0.869		–
Language and understanding	A^a^	19	7.53	0.61	19	7.74	0.56	0.102	17	7.65	0.70	17	7.71	0.69	0.564
	B	20	7.40	1.00	20	7.55	0.83	0.083	20	7.50	0.89	20	7.65	0.67	0.083
	P-value	–	0.923		–	0.607		–	–	0.729		–	0.752		–
Space situation	A^a^	19	0.47	0.51	19	0.47	0.51	1.00	17	0.41	0.51	17	0.41	0.51	1.00
	B	20	0.40	0.50	20	0.40	0.50	1.00	20	0.40	0.50	20	0.45	0.51	0.317
	P-value	–	0.708		–	0.708		–	–	0.964		–	0.845		–
Cognitive impairment (general)	A^a^	19	26.74	2.92	19	28.05	2.01	0.003	17	27.29	2.44	17	27.41	2.57	0.527
	B	20	26.20	4.21	20	26.55	4.10	0.068	20	26.35	4.44	20	27.35	3.53	0.005
	P-value	–	0.901		–	0.461		–	–	0.821		–	0.707		–

^a^The group A took valerian capsules in the first therapeutic period of one month and placebo in the second therapeutic period of one month, and vice versa in group B

In the comparison of the two groups, only the difference in the increased total MMSE score between the two groups in both one-month periods was significant, so that the mean increased MMSE score during the first one-month treatment period was 1.32 ± 1.38 in the Group valerian and 0.35 ± 0.81 in the Group placebo. There was a significant difference in the distribution of scores between the two groups (*P* = 0.028). In the second one-month treatment period, the mean increased total MMSE score was 1.00 ± 1.17 in the Group valerian and 0.12 ± 0.78 in the Group placebo. There was a significant difference in the distribution of scores (*P* = 0.030), so that the increased MMSE score was more in the Group valerian in both periods (Table 3).

**Table 3:** Mean difference of cognitive scores before and after intervention in each treatment periods in the groups

Index	group	First Period			Second Period		
		N	Mean	SD	N	Mean	SD
Orientation	A^a^	19	0.26	0.56	17	0.00	0.00
	B	20	0.10	0.31	20	0.25	0.72
	P-value	0.550			0.442		
Immediate memory	A^a^	19	0.05	0.23	17	0.00	0.00
	B	20	0.10	0.31	20	0.05	0.22
	P-value	0.813			0.798		
Concentration and calculation	A^a^	19	0.47	0.70	17	0.00	0.50
	B	20	0.00	0.32	20	0.30	0.47
	P-value	0.057			0.177		
Remembrance	A^a^	19	0.32	0.48	17	0.06	0.24
	B	20	0.00	0.32	20	0.20	0.41
	P-value	0.113			0.478		
Language and understanding	A^a^	19	0.21	0.53	17	0.06	0.43
	B	20	0.15	0.37	20	0.15	0.37
	P-value	0.728			0.684		
Space situation	A^a^	19	0.00	0.00	17	0.00	0.00
	B	20	0.00	0.00	20	0.05	0.22
	P-value	1.00			0.798		
Cognitive impairment (general)	A^a^	19	1.32	1.38	17	0.12	0.78
	B	20	0.35	0.81	20	1.00	1.17
	P-value	0.028			0.030		

^a^The group A took valerian capsules in the first therapeutic period of one month and placebo in the second therapeutic period of one month, and vice versa in group B

During the first one-month treatment period before the intervention, the EEG was determined for seven patients in the Group A and for eight cases in the Group B; one from the Group B did not cooperate after the intervention. In the second one-month treatment period before the intervention, seven patients in each group collaborated, but after the intervention, only six from Group A and three from Group B continued to cooperate.

In the first one-month treatment period, three out of seven were normal before and after intervention and three had a mild EEG problem before and after intervention. Only one had a mild problem before the intervention, which became normal after intervention; but the changes were not significant (*P* = 1.00). In the Group B, 3 out of 7 people with clear EEG status before and after the intervention had mild problem before intervention, which was mild after the intervention as well, but three people had mild impairment before intervention, which became normal after intervention; one person was normal before intervention that became mild after intervention. The changes were not significant (*P* = 0.625).

In the second one-month treatment period, two out of six in the Group A with detected EEG status had normal status before and after intervention, and one person had a mild state before and after intervention. However, two subjects had a mild state before intervention and became normal after intervention, but one had inversed status who was normal before the intervention and was mild after the intervention; the changes were not significant (*P* = 1.00). In the Group B, of three subjects whose EEG status was determined before and after intervention, two patients were normal before the intervention and one was mild, which all three were normal after intervention (Table 4).

**Table 4:** EEG and type of brain waves of hemodialysis patients before and after in treatments cycles

time	EEG	First Period				Second Period			
		A^a^		B		A^a^		B	
		Number	Percent	Number	Percent	Number	Percent	Number	Percent
Before intervention	Normal (Alpha, Beta)	3	42.9	1	12.5	4	57.1	3	42.9
	Mild (Theta)	4	57.1	7	87.5	3	42.9	4	57.1
After intervention	Normal (Alpha, Beta)	4	57.1	3	42.9	4	66.7	3	100
	Mild (Theta)	3	42.9	4	57.1	2	33.3	–	–

^a^The group A took valerian capsules in the first therapeutic period of one month and placebo in the second therapeutic period of one month, and vice versa in group B

## Discussion

The main findings of this study revealed that valerian could be effective and significantly improve cognitive status although no significant changes were observed in the EGG of the hemodialysis patients. The MMSE scores in the valerian group within the first and the second one-month treatment periods had also significantly increased. In line with these results, Hassani et al. (2013) in Sari, Iran, examined the effects of valerian root extract on early prevention of the postoperative cognitive disorders after coronary artery bypass graft surgery. They conducted a standardized MMSE on the day before surgery, 10 days and 2 months after surgery. A significant reduction in the cognitive disorders was seen in the Group valerian compared to the Group placebo within 10 days after surgery and a greater improvement in cognitive function within eight weeks after surgery [40]. Ceddia et al. (2015) in the United States showed that the extract of herbs from the peppermint family also has a positive effect on cognitive health, including improvement in memory, reasoning, attention, concentration, planning and mood [44]. In the study by Hensel et al. (2007), at least 2–4 points indicated that the reliable changes in the MMSE scores were clinically significant [45]. In the present study, the results showed that valerian could significantly increase the overall cognitive scores of hemodialysis patients (between 0.88 and 0.97). More examples were similarly suggested for the sub-quantifiers that were not significant probably due to the limited time period of the study as well as different times considered for the evaluation of the MMSE scores in morning, evening, and night shifts.

In this study MMSE was completed the first hour of hemodialysis and then the EEG was done in the morning shift on the day after hemodialysis between 8 AM until 12 MD. Drew et al. (2015) also performed cognitive testing during hemodialysis, which in theory may influence cognitive performance through fluid shifts and changes in electrolyte levels [46]. Drew et al. (2013) previously conducted a randomized crossover study in hemodialysis patients found no difference in performance based on the timing of testing [47]. Also, the results of the study by Sperschneider et al. (1980) in Germany showed that changes in EEG remained constant or increased during the hemodialysis period [48]. Also, Wendland and Susantija (1983) in assessment of EEG in the hemodialysis patients before and after hemodialysis, in 10 men and 11 women, showed that an increase in abnormal waves after hemodialysis. The changed of EEG was lower on the day after hemodialysis treatment [49].

In this study, the changes in EEG were not significant in the two groups of A and B in the two one-month treatment periods. Diaper et al. (2004) studied the effects of two different concentrations of valerian on sleep, cognitive function and motor function in elderly people with sleep disorders. The results showed no significant difference between valerian 300 mg, 600 mg or placebo in EEG indices and psychometric measurements [50]. Vonderheid-Guth et al. (2000) in Germany within a double-blind crossover study investigated the pharmacodynamic effects of different amounts of commercial valerian-hops extract mix on quantitative EEG topography (QEEG) in 12 young healthy volunteers compared to the placebo. The results showed that the EEG was able to show mild, but visible, effects especially after taking high concentrations of valerian-hops mix [38].

In this study, the reason for the insignificant changes in EEG before and after intervention might be attributed to inappropriate cooperation of patients with EEG. Some patients refused to perform EEG due to various works and time consuming during the intervention phase. The results of this study can be guidelines for designing new programs and using non-chemical methods to attenuate the cognitive disorders in patients undergoing hemodialysis, as well as can be a model for further researches regarding the examination of other Valerian properties and the use of complementary medicine.

## Conclusion

The present findings demonstrated that valerian was effective as a safe herbal remedy in reducing the cognitive disorders. Regarding the high prevalence of cognitive disorders in hemodialysis patients, the use of valerian for treatment of these disorders may be considered. Additionally, the cognitive function should be investigated in all periodic examinations in these patients in order to provide early diagnosis of the cognitive disorders.

## Abbreviations

BMI: Body mass index
DM: Diabetes mellitus
EEG: Electroencephalography
HD: Heart disease
HTN: Hypertension
MMSE: Mini mental state examination
RLS: Restless leg syndrome

## References

[1] Kalirao P, Pederson S, Foley RN, Kolste A, Tupper D, Zaun D, Buot V, Murray AM (2011). Cognitive impairment in peritoneal dialysis patients. Am J Kidney Dis.

[2] San A, Hiremagalur B, Muircroft W, Grealish L (2017). Screening of cognitive impairment in the Dialysis population: a scoping review. Dement Geriatr Cogn Disord.

[3] Brodaty H, Pond D, Kemp NM, Luscombe G, Harding L, Berman K, Huppert FA (2002). The GPCOG: a new screening test for dementia designed for general practice. J Am Geriatr Soc.

[4] Tamura MK, Larive B, Unruh ML, Stokes JB, Nissenson A, Mehta RL, Chertow GM, Group FHNT. Prevalence and correlates of cognitive impairment in hemodialysis patients: the Frequent Hemodialysis Network trials. Clin J Am Soc Nephrol. 2010;24(5);1–10. 10.2215/CJN.01090210. 10.2215/CJN.01090210 PMC2924414 20576825

[5] Odagiri G, Sugawara N, Kikuchi A, Takahashi I, Umeda T, Saitoh H, Yasui-Furukori N, Kaneko S (2011). Cognitive function among hemodialysis patients in Japan. Ann General Psychiatry.

[6] Grimm G, Stockenhuber F, Schneeweiss B, Madl C, Zeitlhofer J, Schneider B (1990). Improvement of brain function in hemodialysis patients treated with erythropoietin. Kidney Int.

[7] Unruh M, Tamura MK, Larive B, Rastogi A, James S, Schiller B, Gassman J, Chan C, Lockridge R, Kliger A (2011). Impact of sleep quality on cardiovascular outcomes in hemodialysis patients: results from the frequent hemodialysis network study. Am J Nephrol.

[8] Casserly I, Topol EJ (2004). Convergence of atherosclerosis and Alzheimer's disease: inflammation, cholesterol, and misfolded proteins. Lancet.

[9] Eslami Amirabadi M, Hosein DK, Nasrollahi A, Norouzian M, Bozorg B, Kivi A, Mitra S, Salamati SM (2014). Cognitive dysfunction in hemodialysis patients and its related factors. Res Med.

[10] Bae JS, Park SS (2008). Contingent negative variation before and after hemodialysis among patients with end-stage renal disease. J Neurol Sci.

[11] Wilson S, Dhar A, Tregaskis P, Lambert G, Barton D, Walker R (2018). Known unknowns-examining the burden of neurocognitive impairment in the end-stage renal failure population. Nephrology.

[12] Arevalo-Rodriguez I, Smailagic N, Roqué IFM, Ciapponi A, Sanchez-Perez E, Giannakou A, Pedraza OL, Bonfill Cosp X, Cullum S (2015). Mini-mental state examination (MMSE) for the detection of Alzheimer’s disease and other dementias in people with mild cognitive impairment (MCI). Cochrane Database Syst Rev.

[13] Tsoi KK, Chan JY, Hirai HW, Wong A, Mok VC, Lam LC, Kwok TC, Wong SY (2017). Recall tests are effective to detect mild cognitive impairment: A systematic review and meta-analysis of 108 diagnostic studies. J Am Med Dir Assoc.

[14] Drew DA, Weiner DE, Tighiouart H, Duncan S, Gupta A, Scott T, Sarnak MJ (2017). Cognitive decline and its risk factors in prevalent hemodialysis patients. Am J Kidney Dis.

[15] Arsalani N, Nobahar M, Ghorbani R, Kia N, Etemadi M. Cognitive disorders and some associated social factors in elderly pepole. Koomesh. 2018;20(2):240–7.

[16] Sarnak MJ, Tighiouart H, Scott TM, Lou KV, Sorensen EP, Giang LM, Drew DA, Shaffi K, Strom JA, Singh AK (2013). Frequency of and risk factors for poor cognitive performance in hemodialysis patients. Neurology.

[17] Rosenberg S, Perin B, Michel V, Debs R, Navarro V, Convers P (2015). EEG in adults in the laboratory or at the patient's bedside. Neurophysiologie Clinique/Clinical Neurophysiology.

[18] Wen D, Zhou Y, Li X (2015). A critical review: coupling and synchronization analysis methods of EEG signal with mild cognitive impairment. Front Aging Neurosci.

[19] König T, Prichep L, Dierks T, Hubl D, Wahlund L, John E, Jelic V (2005). Decreased EEG synchronization in Alzheimer’s disease and mild cognitive impairment. Neurobiol Aging.

[20] Dauwels J, Vialatte F, Musha T, Cichocki A (2010). A comparative study of synchrony measures for the early diagnosis of Alzheimer's disease based on EEG. NeuroImage.

[21] Sweeney-Reed CM, Riddell PM, Ellis JA, Freeman JE, Nasuto SJ (2012). Neural correlates of true and false memory in mild cognitive impairment. PLoS One.

[22] Tóth B, File B, Boha R, Kardos Z, Hidasi Z, Gaál ZA, Csibri É, Salacz P, Stam CJ, Molnár M (2014). EEG network connectivity changes in mild cognitive impairment—preliminary results. Int J Psychophysiol.

[23] Babiloni C, Ferri R, Binetti G, Cassarino A, Dal Forno G, Ercolani M, Ferreri F, Frisoni GB, Lanuzza B, Miniussi C (2006). Fronto-parietal coupling of brain rhythms in mild cognitive impairment: a multicentric EEG study. Brain Res Bull.

[24] Rohl J, Harms L, Pommer W (2007). Quantitative EEG findings in patients with chronic renal failure. Eur J Med Res.

[25] Griva K, Thompson D, Jayasena D, Davenport A, Harrison M, Newman SP (2006). Cognitive functioning pre-to post-kidney transplantation—a prospective study. Nephrol Dial Transplant.

[26] Kallenberg MH, Kleinveld HA, Dekker FW, van Munster BC, Rabelink TJ, van Buren M, Mooijaart SP. Functional and cognitive impairment, frailty, and adverse health outcomes in older patients reaching ESRD—a systematic review. Clin J Am Soc Nephrol. 2016;24(11):1–16. 10.2215/CJN.13611215. 10.2215/CJN.13611215 PMC5012494 27342598

[27] Kooman JP, van der Sande FM, Leunissen KM (2017). Kidney disease and aging: a reciprocal relation. Exp Gerontol.

[28] Bossola M, Antocicco M, Di Stasio E, Ciciarelli C, Luciani G, Tazza L, Rosa F, Onder G (2011). Mini mental state examination over time in chronic hemodialysis patients. J Psychosom Res.

[29] Shimazaki M, Martin JL (2007). Do herbal agents have a place in the treatment of sleep problems in long-term care?. J Am Med Dir Assoc.

[30] Yao M, Ritchie HE, Brown-Woodman PD (2007). A developmental toxicity-screening test of valerian. J Ethnopharmacol.

[31] Taibi DM, Vitiello MV, Barsness S, Elmer GW, Anderson GD, Landis CA (2009). A randomized clinical trial of valerian fails to improve self-reported, polysomnographic, and actigraphic sleep in older women with insomnia. Sleep Med.

[32] Dimpfel W, Brattstrom A, Koetter U (2006). Central action of a fixed valerian-hops extract combination (ZE 91019) in freely moving rats. Eur J Med Res.

[33] Sharma M, Jain U, Patel A, Gupta N (2010). A comprehensive pharmacognostic report on valerian. Int J Pharm Sci Res.

[34] Gyllenhaal C, Merritt SL, Peterson SD, Block KI, Gochenour T (2000). Efficacy and safety of herbal stimulants and sedatives in sleep disorders. Sleep Med Rev.

[35] Jariani M, Saki M, Saki K, Ahmadi H, Roohandah M, Tarahi M (2009). Effectiveness of valerian as a complementary medicine on bipolar mood disorders. J Ilam Univ Med Sci.

[36] Sampath C, Haug K, Thanei S, Hamburger M, Derendorf H, Frye R, Butterweck V (2012). Pharmacokinetics of valerenic acid in rats after intravenous and oral administrations. Planta Med.

[37] Zarei A, Ashtiyani SC, Hamidizadeh S, Rezaei A (2015). The study of the effects hydro-alcoholic extract of Eryngium billardieri on lipid profiles levels and liver and renal functions tests in hypercholesterolemic rats. Global J Pharmacol.

[38] Vonderheid-Guth B, Todorova A, Brattström A, Dimpfel W (2000). Pharmacodynamic effects of valerian and hops extract combination (Ze 91019) on the quantitative-topographical EEG in healthy volunteers. Eur J Med Res.

[39] Schulz H, Jobert M, Hübner W (1998). The quantitative EEG as a screening instrument to identify sedative effects of single doses of plant extracts in comparison with diazepam. Phytomedicine.

[40] Hassani S, Alipour A, Darvishi Khezri H, Firouzian A, Emami Zeydi A, Gholipour Baradari A, Ghafari R, Habibi W, Tahmasebi H, Alipour F. Can *Valeriana officinalis* root extract prevent early postoperative cognitive dysfunction after CABG surgery. A randomized, double-blind, placebo-controlled trial. Psychopharmacology (Berl). 2015:232(5);843–50. 10.1007/s00213-014-3716-x 25173770

[41] O’Lone E, Connors M, Masson P, Wu S, Kelly PJ, Gillespie D, Parker D, Whiteley W, Strippoli GF, Palmer SC (2016). Cognition in people with end-stage kidney disease treated with hemodialysis: a systematic review and meta-analysis. Am J Kidney Dis.

[42] Laudański K, Nowak Z, Wańkowicz Z (2010). Psychological aspect of dialysis: does cognitive appraisal determine the overall outcome. Pol Arch Med Wewn.

[43] Afshar R, Sanavi S, Salimi J (2007). Epidemiology of chronic renal failure in Iran: a four year single center experience. Saudi J Kidney Dis Transplant.

[44] Ceddia M, Herrlinger K, Lewis B, Feng S, Nieman K: Plant extracts for improving cognitive function. In.: Google Patents; 2015. patents.google.com/patent/US20160166629A1/en.

[45] Hensel A, Angermeyer MC, Riedel-Heller SG. Measuring cognitive change in older adults: reliable change indices for the MMSE. J Neurol Neurosurg Psychiatry. 2007;18:1–15. 10.1136/jnnp.2006.109074. 10.1136/jnnp.2006.109074 PMC2095596 17442763

[46] Drew DA, Weiner DE, Tighiouart H, Scott T, Lou K, Kantor A, Fan L, Strom JA, Singh AK, Sarnak MJ (2015). Cognitive function and all-cause mortality in maintenance hemodialysis patients. Am J Kidney Dis.

[47] Drew DA, Tighiouart H, Scott TM, Lou KV, Shaffi K, Weiner DE, Sarnak MJ (2013). Cognitive performance before and during hemodialysis: a randomized cross-over trial. Nephron Clin Pract.

[48] Sperschneider H, Stein G, Mühlau G, Both R, Fünfstück R, Wieczorek V (1980). EEG and ENG findings in patients with chronic renal insufficiency and in hemodialysis patients. Zeitschrift fur die gesamte innere Medizin und ihre Grenzgebiete.

[49] Wendland K, Susantija T (1983). EEG studies before and after hemodialysis. Klin Wochenschr.

[50] Diaper A, Hindmarch I (2004). A double-blind, placebo-controlled investigation of the effects of two doses of a valerian preparation on the sleep, cognitive and psychomotor function of sleep-disturbed older adults. Phytother Res.

